# Fault Feature Extraction for Reciprocating Compressors Based on Underdetermined Blind Source Separation

**DOI:** 10.3390/e23091217

**Published:** 2021-09-15

**Authors:** Jindong Wang, Xin Chen, Haiyang Zhao, Yanyang Li, Zujian Liu

**Affiliations:** 1 Mechanical Science and Engineering Institute, Northeast Petroleum University, Daqing 163318, China; nepusyjxgc@nepu.edu.cn (J.W.); chenxinnpu@gmail.com (X.C.); yanyangli327@gmail.com (Y.L.); zujianliu327@gmail.com (Z.L.); 2 Heilongjiang Key Laboratory of Petroleum Machinery Engineering, Daqing 163318, China

**Keywords:** underdetermined blind source separation, mixing matrix estimation, K-means, reciprocating compressor, feature extraction

## Abstract

In practical engineering applications, the vibration signals collected by sensors often contain outliers, resulting in the separation accuracy of source signals from the observed signals being seriously affected. The mixing matrix estimation is crucial to the underdetermined blind source separation (UBSS), determining the accuracy level of the source signals recovery. Therefore, a two-stage clustering method is proposed by combining hierarchical clustering and K-means to improve the reliability of the estimated mixing matrix in this paper. The proposed method is used to solve the two major problems in the K-means algorithm: the random selection of initial cluster centers and the sensitivity of the algorithm to outliers. Firstly, the observed signals are clustered by hierarchical clustering to get the cluster centers. Secondly, the cosine distance is used to eliminate the outliers deviating from cluster centers. Then, the initial cluster centers are obtained by calculating the mean value of each remaining cluster. Finally, the mixing matrix is estimated with the improved K-means, and the sources are recovered using the least square method. Simulation and the reciprocating compressor fault experiments demonstrate the effectiveness of the proposed method.

## 1. Introduction

With the rapid development of modern industry and technology, mechanical equipment is growing larger and more precise with high-speed [1]. Any fault of the equipment can possibly cause a breakdown of the entire mechanical system, which may cause the interruption of production, huge economic losses, and even casualties. Many mechanical fault diagnosis methods have been proposed to ensure the normal and stable operation of equipment, such as oil analysis, acoustic emission detection, temperature detection, and vibration analysis. The advantage of vibration analysis is its ability to retain abundant fault information, and thus is widely used in condition monitoring and feature extraction of mechanical equipment [2].

Reciprocating compressor are commonly used in the petroleum, chemical, and transportation industry with the advantages of wide pressure distribution and high compression efficiency. Therefore, how to maintain the normal operation of reciprocating compressor has attracted considerable attention in recent years [3,4,5]. Due to the complex structure of the reciprocating compressor, numerous internal excitation sources, and the mutual coupling of each source signal, the collected vibration signals mostly present non-stationarity and nonlinearity characteristics. Traditional signal analysis methods such as empirical mode decomposition (EMD) [6,7], local mean decomposition (LMD) [8,9], variational mode decomposition (VMD) [10,11] have been successfully applied in the field of rotating machinery fault diagnosis. However, these methods may fail to extract effective features from the vibration signals in the fault diagnosis procedures of reciprocating compressor [1].

Blind source separation (BSS) is an advanced signal processing technique that can accurately separate the source signals from their mixtures without relying on prior knowledge, which has wide applications in various areas such as biomedical engineering [12,13], speech recognition [14,15], and fault diagnosis [16,17]. Recently, BSS algorithms have been rapidly developed and have been successfully applied in real engineering applications. For example, Zhou et al. [18] combined independent component analysis (ICA) and principal component analysis for blast furnace ironmaking condition monitoring and fault diagnosis. To extract the weak fault feature of rotating machinery from the observed signals, Miao et al. [19] proposed a BSS method based on fast independent component analysis (Fast ICA) and wavelet packet analysis. He et al. [20] studied the weak characteristic determination of centrifugal compressors with underdetermined blind source separation (UBSS) based on sparse component analysis (SCA) and successfully detected the cracked blade information from the pressure pulsation. However, the above studies mainly focus on the positive or overdetermined cases, i.e., the number of sensors is greater than or equal to the number of sources, but this constraint is difficult to be met in practical engineering applications. Moreover, much research methods based on BSS or UBSS have focused on rotating machinery and seldom literature has considered the fault diagnosis on the reciprocating compressor. Therefore, we proposed a new UBSS method and apply it to the fault feature extraction of the reciprocating compressor.

Sparse component analysis, as a common UBSS method, can separate the source signals by exploiting the sparsity characteristics of sources in the transform domain [21,22]. Generally, the SCA algorithm consists of two steps: mixing matrix estimation and source recovery [23]. Among the two-step approach, the mixing matrix estimation has been widely considered to be the most important step [15,24,25]. The potential function and clustering are the two most common methods used to estimate the mixing matrix. The potential function can estimate the mixing matrix without knowing the number of the sources. For example, Bofill et al. [26] firstly proposed the potential function method to estimate the mixing matrix to separate the flute signals from the mixing signals. Hao et al. [27] further extended the potential function to three-dimensional space and achieved good results in separating bearing compound fault signals and extracting the fault feature. However, in summary, these methods based on potential function are limited in how many sensors they can use. In contrast, the clustering methods, such as hierarchical clustering [28,29], Fuzzy C-means (FCM) clustering [30,31], K-means clustering [32,33], can get rid of this limitation by allowing an arbitrary number of sensors. The clustering methods mentioned above have drawn tremendous interest and are popularly used for the mixing matrix estimation in the SCA. As an illustration, Lu et al. [34] applied hierarchical clustering to estimate the mixing matrix of the cylindrical structure fault signals, and thus identified the major vibration of the mechanical systems. Hu et al. [35] applied the FCM and L1 norm to extract the fault feature of the wind turbine gearbox, which successfully detected the fault location. He et al. [36] proposed an improved K-means by combining the Laplace potential function to estimate the mixing matrix of bearing vibration signals.

In summary, although these methods can all estimate the mixing matrix, there are still some accuracy level limitations in the source recovery as reported in [28,36]. In contrast to hierarchical clustering and FCM clustering, the K-means method has a fast computation speed and relatively higher estimation accuracy. However, the clustering accuracy is usually unstable because its initial clustering centers are generated randomly. To overcome the drawback that K-means is sensitive to the initial clustering centers, Xie et al. [37] employed the firefly optimization algorithm (FOA) to optimize the initial clustering centers, and thus achieved enhanced centers with fast convergence. Arthur et al. [38] proposed a novel K-means++ algorithm based on K-means to solve the above-mentioned problem. In addition, He et al. [39] applied the K-means to estimate the mixing matrix by combining the affinity propagation (AP) clustering to preset the initial clustering of K-means, which can avoid the random selection. The above various improved K-means algorithms have achieved great results in the mixing matrix estimation. However, another challenge in the K-means is that the outliers existing in the observed signals may have a significant influence on clustering, which has been ignored. To alleviate the effect of these outliers, Reju et al. [40] firstly proposed a quadratic hierarchical clustering method to remove the outliers existing in the clusters. More specifically, the initial clustering centers are obtained by the first hierarchical clustering, the cosine distance is used to eliminate the outliers deviating from the initial clustering centers, and then the remaining clusters are clustered again by the second hierarchical clustering.

Inspired by the idea of quadratic clustering and integrating the advantages of K-means, a two-stage clustering method is proposed by combining hierarchical clustering and K-means. The purpose of this study is to solve the two main problems in the K-means algorithm: the random selection of initial cluster centers and the sensitivity of the algorithm to outliers. Firstly, the hierarchical clustering method is used to cluster the mixed signals to obtain the clustering centers, and some outliers deviating from the clustering center are eliminated. Then, the new clustering centers obtained by calculating the mean value of the remaining clusters are set as the initial clustering centers of K-means. Finally, the source signals are separated with the least square method. The simulation and reciprocating compressor experiments prove the effectiveness of the proposed method.

The rest part of the paper is organized as follows: Section 2 introduces the UBSS method based on SCA. Section 3 states the problem existing in the K-means and presents the proposed mixing matrix estimation method by combining hierarchical clustering and K-means clustering. Section 5 presents a simulation analysis, a comparison with the traditional K-means, and the application in reciprocating compressor fault diagnosis and draws conclusions.

## 2. Underdetermined Blind Source Separation Based on SCA

### 2.1. Basic Theory of UBSS

The linear instantaneous mixed model of UBSS is shown as follows:

(1) x(t)=As(t)

where $x(t)={\left[x_{1}(t),x_{2}(t),\cdots ,x_{m}(t)\right]}^{T}$ is an *m*-dimensional observed signal, *x_i_* is the observation value of the *i*-th sensor at time point *t*. $A=[a_{1},a_{2},\cdots ,a_{n}]\in {\mathbb{R}}^{m\times n}$ is a mixing matrix, $a_{i}$ is the *i*-th column vector of the matrix A. $s(t)={\left[s_{1}(t),s_{2}(t),\cdots ,s_{n}(t)\right]}^{T}$ is an *n*-dimensional source signal, and *s_i_*(*t*) is the sample point of the *i*-th source at time point *t*. *m* and *n* are the number of the observed signal and source signals, respectively. However, this study focuses on the UBSS under the condition that n>m≥2, which is more suitable for engineering applications.

To obtain the sparse signals, the mixed model in Equation (1) is transformed to the time-frequency (TF) domain with the short-time Fourier transform (STFT) [41,42]. The process is described as:

(2) $$x(t,f)=As(t,f)=\sum_{i=1}^{n}a_{i}s_{i}(t,f)$$

where **x**(*t*) and **s**(*t*) are the TF coefficients of **x**(*t,f*) and **s**(*t,f*), respectively.

**Figure 1:**
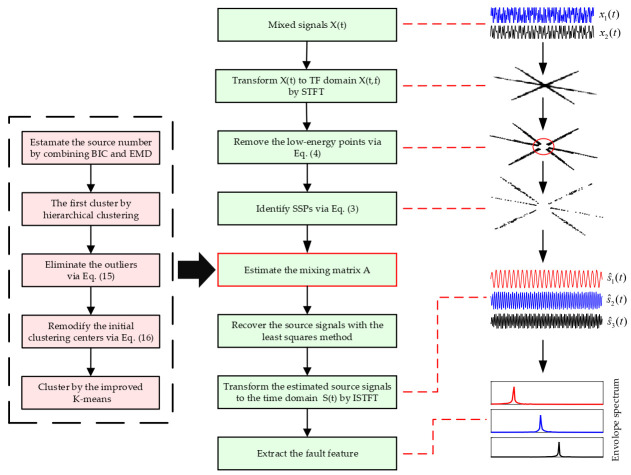
The flow chart of fault feature extraction based on underdetermined blind source separation.

After obtaining the sparse signals **x**(*t,f*), the UBSS based SCA algorithm can be employed to recover the source signals. The detailed steps of the UBSS-SCA are depicted in Figure 1.

### 2.2. Single Source Points Detection

The single source points (SSPs) detection is used as a preprocessing before the mixing matrix estimation for enhancing the sparse representation of the source signals in the TF domain and thus improving the accuracy of the estimated mixing matrix. In the literature [40], an SSPs identification method was proposed by comparing the directions of the absolute values of the real and imaginary parts of the observed signals by:

(3) $$\left|\frac{R{\left\{x(t,f)\right\}}^{T}I\{x(t,f)\}}{{\left\|R\{x(t,f)\}\right\|}_{2}{\left\|I\{x(t,f)\}\right\|}_{2}}\right|\;<\cos(\Delta \theta )$$

where $\left|\cdot \right|$ represents the absolute value and Δθ is a threshold usually set to be close to 0, the literature [26] suggested ∆θ=0.2∼0.8.

Since noise may interfere with the sparse representation of observed signals in the TF domain, so it is necessary to effectively reduce the strength of interference by eliminating the low-energy mixed TF vectors if:

(4) $${\left\|x(t,f)\right\|}_{2}<\varepsilon \cdot \max\left\{{\left\|x(1,1)\right\|}_{2},\ldots ,{\left\|x(T,K)\right\|}_{2}\right\}$$

Note that, in (4), *T* and *K* represent the number of time and frequency points, respectively, and ε represents a fraction number, commonly set to be 0.01.

### 2.3. Mixing Matrix Estimation Based on K-Means

K-means is an unsupervised learning clustering algorithm with the advantages of fast computation and high accuracy, which is widely used for the mixing matrix estimation in UBSS. The principle is described as follows [43].

Given a sample set $X=\{x_{1},x_{2},\cdots ,x_{n}\}$, randomly select an initial cluster center $\left(m_{1},m_{2},\cdots ,m_{k}\right)$, and find a partition *C* such that the objective function is minimized by:

(5) $$\underset{C}{\min}\sum_{l=1}^{k}\sum_{C(i)=l}{\left\|x_{i}-m_{l}\right\|}^{2}$$

where $\left\|\cdot \right\|$ denotes the euclidean norm and *k* is the number of clusters. 

Then, for a given division *C*, the center $\left(m_{1},m_{2},\cdots ,m_{k}\right)$ of each cluster is found, such that the objective function is minimized by:

(6) $$\underset{m_{1},m_{2},\cdots ,m_{k}}{\min}\sum_{l=1}^{k}\sum_{C(i)=l}{\left\|x_{i}-m_{l}\right\|}^{2}$$

For each class $G_{l}$ containing *n_i_* samples, update its mean value *m_l_*:

(7) $$m_{l}=\frac{1}{n_{l}}\sum_{C(i)=l}x_{i},l=1,2,\cdots ,k$$

Repeat the above steps until the cluster center *m_l_* does not change anymore. The obtained clustering center $\left(m_{1},m_{2},\cdots ,m_{k}\right)$ is the estimated mixing matrix $\hat{A}$.

### 2.4. Source Recovery

After obtaining the estimated mixing matrix $\hat{A}$, the sources are recovered by solving a series of least square problems [44]. Specifically, A is defined as m×(m−1) sub-matrices of $\hat{A}$, which is represented by:

(8) $$A=\{A_{i}|A_{i}=[a_{\theta_{1}},a_{\theta_{2}},\ldots ,a_{\theta_{m-1}}]\}$$

Obviously, A obtains $C_{n}^{\left(m-1\right)}$ elements, i.e., $i=1,2,\cdots ,C_{n}^{\left(m-1\right)}$.

For any given TF point (t,f), there is a matrix $A_{*}=[a_{\phi_{1}},a_{\phi_{2}},\ldots ,a_{\phi_{m-1}}]$ in the set A, which satisfies (9):

(9) $$\tilde{x}(t,k)=A_{*}A_{*}^{†}\tilde{x}(t,k)$$

where $A_{*}^{†}$ is the pseudo-inverse of $A_{*}$.

Then, the source signals can be recovered through the following formula:

(10) $${\hat{s}}_{j}(t,k)=\;\left\{\begin{array}{ll} e_{i}, & \;\mathrm{if}\;j=\phi_{i}\\ 0, & \;\mathrm{otherwise}\; \end{array}\right.$$

where $e={\left[e_{1},e_{2},\cdots ,e_{m-1}\right]}^{T}=A_{*}^{†}\hat{x}(t,f)$, and $A_{*}$ can be obtained by:

(11) $$A_{*}=\arg\underset{A_{i}\in A}{\min}∥\tilde{x}(t,k)-A_{i}A_{i}^{†}\tilde{x}(t,k){∥}_{2}$$

Finally, the estimated TF source signal $\hat{s}(t,f)$ is transformed back into the time domain by inverse short-time Fourier transform (ISTFT), which provides the time domain estimated signals $\hat{s}(t)$ as required. 

## 3. The Proposed Mixing Matrix Estimation Method

### 3.1. The Statement of K-Means Clustering

From the principle of K-means described in Section 2.3, we can find that there are two major problems in K-means. The one is that the K-means algorithm is sensitive to outliers. When some outliers, such as noise, exist in the original sample sets, the K-means will take these outliers as target data points, and divide them into the nearest clusters from the initial clustering center during the clustering process. Then, the initial clustering center will be updated based on the obtained clusters. Therefore, the clustering accuracy level will decrease in the iteration process because of the existence of outliers. The other is that the random selection issue of the initial clustering centers. From Equation (5), we can find that the initial clustering centers are randomly generated and thus the sets are divided into *k* clusters according to the given clustering centers. As we know, K-means is a partition-based clustering algorithm whose results greatly depend on the selection of the initial clustering centers. Once the clustering centers are set, the iteration will be executed according to the original clustering centers, which cannot guarantee that the solution is the global optimal.

To address the above challenging issues, a novel cluster algorithm is proposed by combining hierarchical clustering and K-means. Unlike FCM and K-means, hierarchical clustering does not have the problem of a random selection of the initial clustering centers and its result is relatively stable. Therefore, it is chosen as the first cluster in this study to get the original clustering centers, and then cosine distance was used to eliminate part outliers deviating from that clustering centers. Moreover, aiming at the random selection problem of the initial clustering centers in K-means, the revised clustering centers after removing the outliers are set as the initial clustering centers. Finally, the improved K-means algorithm is employed to estimate the mixing matrix.

### 3.2. Sources Number Estimation Based on EMD and BIC

Before the mixing matrix estimation, the number of clusters (the source number) needs to be predetermined. The Bayesian information criterion (BIC) is robust to non-Gaussian sources, and thus it can be used to estimate the number of mechanical vibration sources in this study. 

The objective of the Minaka Bayesian selection model (MIBS) based on BIC is to find a number k=n (1≤k≤l) that maximizes the cost function. The cost function of the MIBS model is as follows [45]:

(12) $$MIBS(k)=p(x|k)\approx p_{k}{\left(\sum_{j=1}^{k}\lambda_{j}\right)}^{-N/2}{\tilde{\sigma }}_{k}^{-N(l-k)}{\left|A_{k}\right|}^{-1/2}{\left(2\pi \right)}^{\left(d_{k}+k\right)/2}N^{-k/2}$$

Ignoring all terms that do not grow with *N*, MIBS can be approximated by the BIC [40]:

(13) $$BIC(k)={\left(\prod_{j=1}^{k}\lambda_{j}\right)}^{-N/2}{\tilde{\sigma }}_{k}^{-N(l-k)/2}N^{-\left(d_{k}+k\right)/2}$$

where *N* is the length of the data, *l* is the number of non-zero eigenvalues.

The BIC requires the number of mixed signals *m* must be larger than the number of source signals *n*. To address this problem in UBSS, the EMD decomposition algorithm is introduced in this study to construct a virtual multi-channel sensor. EMD, as an adaptive nonlinear and nonstationary signal analysis method, can decompose a complex single-component signal into a finite number of Intrinsic Mode Function (IMF). For the detailed theory of EMD, please refer to the reference [6].

The steps based on EMD and BIC to estimate the source number are as follows:

Step 1: EMD decomposition. A single channel signal $x_{i}$ in mixed signal $x(t)={\left[x_{1}(t),x_{2}(t),\cdots ,x_{m}(t)\right]}^{T}$ is selected as the EMD decomposition object to obtain a multi-channel signal $x_{IMF}(t)={\left[c_{1},c_{2},\cdots ,c_{n-1},c_{n}\right]}^{T}$, *n* is the number of IMF.
Step 2: Calculate the covariance matrix. The covariance matrix of $x_{IMF}$ is calculated by $R_{IMF}=E[x_{IMF}(t)x_{IMF}^{H}(t)]$.
Step 3: Singular value decomposition. Compute the singular values of $R_{IMF}$ to obtain *M* non-zero singular values in descending order $\Lambda =\{\lambda_{1},\lambda_{2},\cdots ,\lambda_{M}\}$.
Step 4: Sources number estimation. The BIC(*k*) (*k* = 1, 2, …, *M*) of each eigenvalue is calculated, and the *k* corresponding to the largest BIC value is used as the number of the estimated sources.

### 3.3. Mixing Matrix Estimation by Combining Hierarchical Clustering and K-Means

First, the mixed signals are normalized and mapped to the upper-right half hypersphere:

(14) $$\tilde{x}(t,f)=\frac{x(t,f)}{{\left\|x(t,f)\right\|}_{2}}\times sign(x_{1}(t,f))$$

Then, the mixed signal $\tilde{x}(t,f)$ is clustered by the hierarchical clustering algorithm for the first time to obtain the cluster center $C_{first}\;{=[c}_{1}{,c}_{2},\cdots {,c}_{k}]$ and its corresponding cluster $G_{first}\;{=[g}_{1}{,g}_{2},\cdots {,g}_{k}]$. Note that $c_{k}$ represents the cluster center corresponding to the cluster $g_{k}$, and *k* represents the number of clusters.

Due to the influence of outliers such as noise, the computed cluster may deviate from the true cluster center. Therefore, the pairwise cosine distances *dc* between all data points in each cluster and its corresponding cluster center are calculated, and the data points that satisfy the following condition in (15) are taken to be outliers and are thus removed:

(15) $$dc=1-\frac{c_{i}\cdot g_{i}}{{\left\|c_{i}\right\|}_{2}{\left\|g_{i}\right\|}_{2}}>\Delta \omega ,i=1,2,\cdots ,k$$

where Δω is a threshold, typically set to be close to zero. Clearly, the smaller the threshold Δω is, the more data points will be taken to be outliers and removed.

The new cluster center $C_{new}\;{=[c}_{new1}{,c}_{new2},\cdots {,c}_{newk}]$ is obtained by calculating the mean value of each cluster $G_{new}=\;[g_{new1},\;g_{new2},\;\cdots ,\;g_{newk}]$ after removing the outliers:

(16) $$c_{newi}=mean(g_{newi}),\;i=1,2,\cdots ,k$$

Finally, C*_new_* is used as the initial clustering center of K-means, and the second clustering is performed by the improved K-means to get the clustering center C*_second_*, i.e., the estimated mixing matrix $\hat{A}$. The procedure of the proposed method is summarized in Algorithm 1.
**Algorithm 1** The procedure of the proposed mixing matrix estimation algorithm.**Input:** The observed signals x(t), the sources number *k,* the constraint parameter Δω.1:  Transform the observed signals x(t) into the TF domain using STFT to obtain the sparse signal x(t,f).2:  Normalize x(t,f) by Equation (14).3:  Hierarchical clustering is used to cluster the normalized data to obtain the cluster center $C_{first}$ and cluster $G_{first}$.4:  Remove the outliers deviating from the clustering center $C_{first}$ via Equation (15) to get the new center $G_{new}$.5:  The cluster center $C_{second}$ is obtained by calculating the mean value of each cluster $g_{newi}$ via Equation (16).6:  Cluster by setting $C_{new}$ as the initial clustering center of K-means.7:  Calculate the cluster centers of *k* clusters as the estimated mixing matrix $\hat{A}$.**Output:** The estimated mixing matrix $\hat{A}$.

Combining the description of UBSS-SCA in Section 2, the proposed method is shown by the flowing flowchart.

## 4. Application Cases

### 4.1. Simulation Analysis

**Figure 2:**
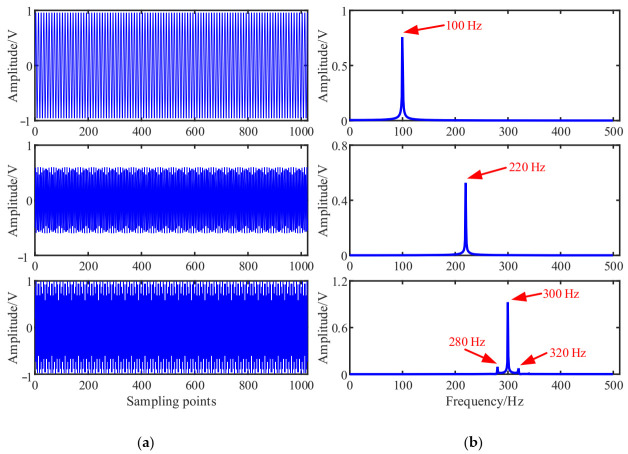
Source signals: (**a**) Waveforms; (**b**) Fourier spectrums.

The simulated source signal $s={\left[s_{1},s_{2},s_{3}\right]}^{T}$ is constructed to verify the effectiveness of the proposed method, as shown in Equation (17) with the sampling frequency of 1000 Hz and sampling points of 1024. In all the experiments, the window length is 512, the number of overlapped samples is 508 and the Hanning window is selected as the window function, the SSPs threshold ∆θ=0.6 and the threshold ∆ω=0.0001. The time domain waveform and Fourier spectrums of the three source signals are shown in Figure 2.

(17) $$\left\{\begin{array}{l} s_{1}=\sin(2\pi f_{1}t)\\ s_{2}=0.6*\cos(2\pi f_{2}t+10)\\ s_{3}=\sin(2\pi f_{3}t+0.2*\cos(2\pi f_{r}t) \end{array}\right.$$

We note that, $f_{1}=100\mathrm{Hz}$, $f_{2}=220\mathrm{Hz}$, $f_{3}=300\mathrm{Hz}$, and $f_{r}=20\mathrm{Hz}$.

A random mixing matrix **A** is generated to obtain the mixed signal **x**(*t*) as follows:

(18) $$A=\left[\begin{array}{rrr} 0.6986 & 0.5575 & 0.9295\\ 0.7155 & -0.8301 & -0.3688 \end{array}\right]$$

**Figure 3:**
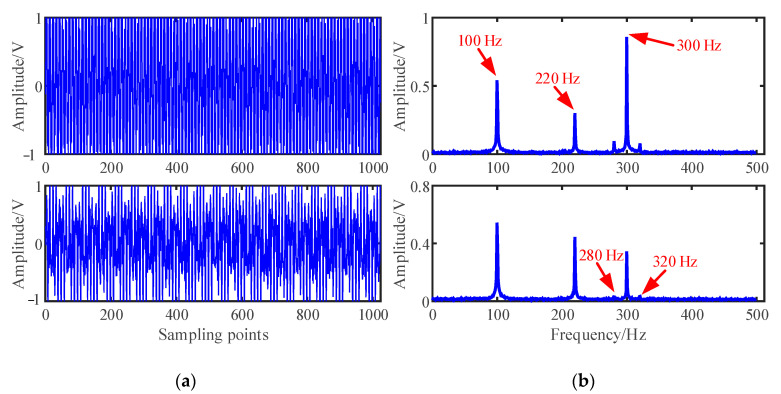
Mixed signals: (**a**) Waveforms; (**b**) Fourier spectrums.

The mixed signal **x**(*t*) is constructed according to Equation (1), and 10% root mean square (RMS) Gaussian white noise with mean 0 and standard deviation 0.1 generated by a random function is added to the mixed signals. The time domain waveforms and Fourier spectrums of the mixed signal are shown in Figure 3:

(19) x(t)=A∗s(t)+Noise

As shown in Figure 3, the three source signals are completely mixed in the mixed signal *x*_1_ and *x*_2_, and the waveforms of source signals in Figure 2a cannot be identified. In the spectra of mixed signals, the frequency components interfere with each other. It is also obvious that the 280 Hz and 320 Hz modulation frequency components are completely submerged in the spectrum of the mixed signal *x*_2_.

Firstly, the BIC is used to estimate the number of source signals. The mixed signal *x*_1_ is selected as a single channel signal and input to EMD for decomposition to obtain 9 modal components, and the multi-channel signal is constructed. Secondly, the covariance matrix of *x_imf_* is calculated, and SVD is used to decompose the matrix to obtain the singular values in descending order, as shown in Table 1.

**Figure 4:**
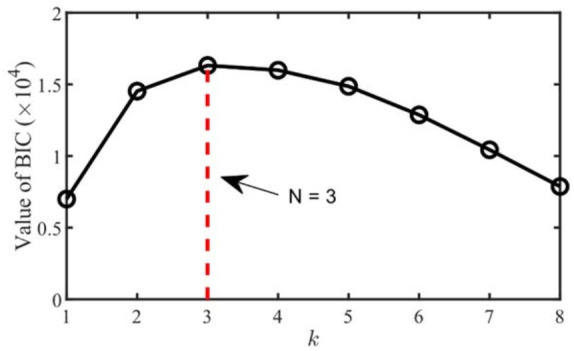
Source number estimation by BIC.

Then, the eigenvalues are input to Equation (13), and the results are shown in Figure 4. When *k* = 3, the corresponding BIC value is the largest. Therefore, the number of source signals can be determined to be 3. The result is consistent with the number of source signals in Figure 2.

**Figure 5:**
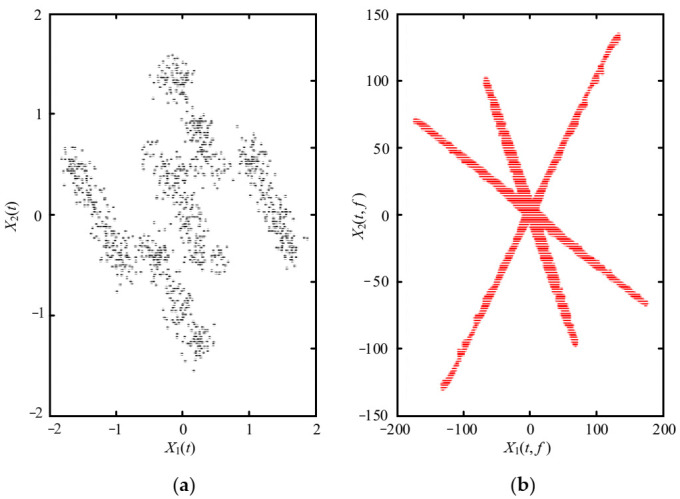
The scatter plot of mixed signals: (**a**) Time domain; (**b**) TF domain by STFT.

The mixed signals are transformed into the TF domain using the STFT, and the scatter plot is shown in Figure 5. It can be observed from Figure 5 that the signals present strong linear characteristics in the TF domain, while the distribution of scatter plots in the time domain is chaotic. The mixed signals in Figure 5b are approximately clustered into three straight lines, indicating that the mixed signals have stronger sparsity in the TF domain than in the time domain.

**Figure 6:**
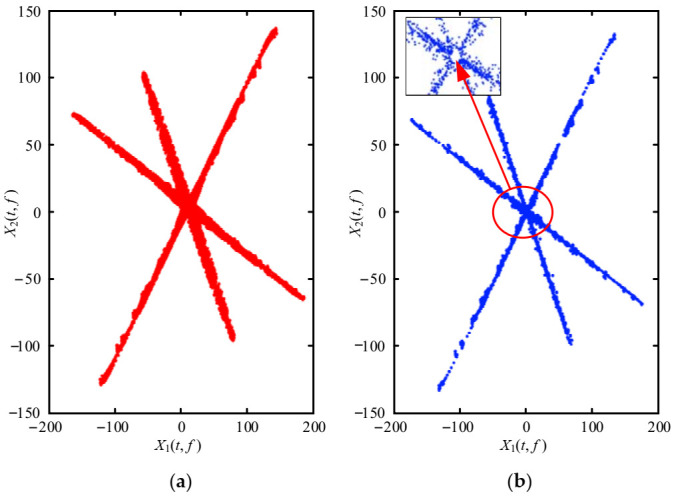
The scatter plot of mixed signals in TF domain: (**a**) Before SSPs; (**b**) After SSPs.

The SSPs are selected from the TF points to enhance the scatter plot’s linear characteristics and improve the accuracy of the estimated mixing matrix. Figure 6b is the scatter plot after SSPs processing. The linear characteristics between the mixed signals are clearer in Figure 6b after identifying the SSPs than in Figure 6a. Besides, there is no overlap between the frequency points in Figure 6a,b, which is beneficial for clustering.

**Figure 7:**
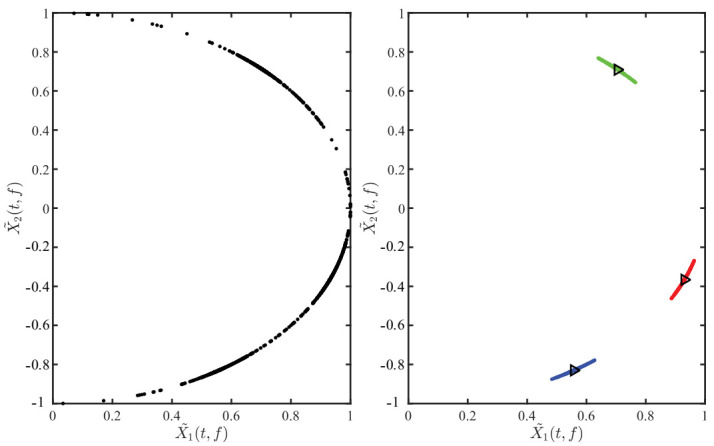
Normalized TF scatter plot: (**a**) Scatter plot before processing; (**b**) Scatter plot after using cosine distance to remove the outliers based on (**a**).

After identifying the SSPs, the TF points are mapped to the upper right half hypersphere by Equation (14), and the normalized TF scatter plot is shown in Figure 7a. The boundary of the three clusters is not obvious, causing a great interference to cluster. Therefore, the outliers in Figure 7a are removed by Equation (15), and the obtained scatter plot is shown in Figure 7b. The clusters in Figure 7b are more compact than Figure 7a, and the boundary between each is clear.

The final estimated mixing matrix is obtained as follows:

(20) $$\hat{A}=\left[\begin{array}{rrr} 0.7032 & 0.5566 & 0.9298\\ 0.7110 & -0.8308 & -0.3680 \end{array}\right]$$

The difference between the absolute values of the mixing matrix **A** and the estimated mixing matrix $\hat{A}$ is shown in Equation (21), indicating that the estimated mixing matrix is very close to the original mixing matrix and the clustering accuracy of the proposed method is very high:

(21) $$\left|A-\hat{A}\right|\;=\;\left[\begin{array}{rrr} 0.0046 & 0.0009 & 0.0003\\ 0.0045 & 0.0007 & 0.0008 \end{array}\right]$$

**Figure 8:**
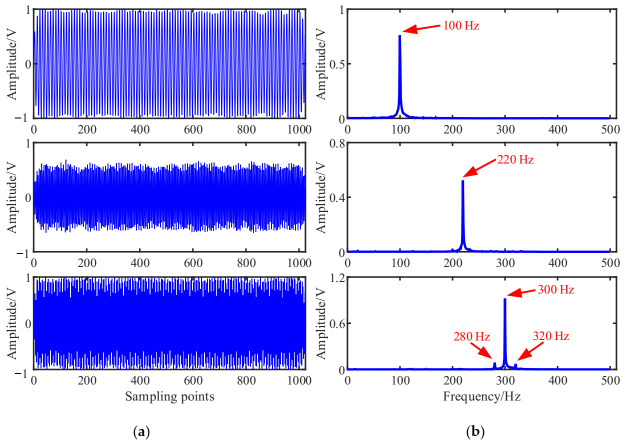
Estimated source signals: (**a**) Waveforms; (**b**) Fourier spectrums.

After the mixing matrix is accurately obtained, the source signal is recovered by the least square method, and time domain waveforms and spectrums of the estimated source signals are shown in Figure 8. The results are obtained by calculating the correlation coefficients of the time domain waveforms in Figure 8a and Figure 2a, and the values are 0.9981, 0.9960, and 0.9981, which indicates that the estimated source signal $\hat{s}\left(t\right)$ is very similar to the source signal **s**(*t*). The Fourier spectrums in Figure 8b show that the main frequencies of the source signal **s**(*t*) can be recovered accurately, thus verifying the effectiveness of the proposed method.

### 4.2. Comparision with the Traditional K-Means

To confirm the proposed method has superiority in the mixing matrix estimation and source signals, the traditional K-means algorithm is used in this study. As a comparison, the mixed signals in Figure 3 are also analyzed by the K-means. The estimated mixing matrix is given as follows:

(22) $$\hat{A}=\left[\begin{array}{rrr} 0.7043 & 0.5703 & 0.9332\\ 0.7099 & -0.8214 & -0.3592 \end{array}\right]$$

Normalized mean square error (NMSE) and the deviation angle are used to evaluate the accuracy of the mixing matrix estimation [40,46], and the calculation formulas are shown in Equations (23) and (24), respectively:

(23) $$\mathrm{NMSE}=10\log_{10}\left(\frac{\sum_{i=1}^{m}\sum_{j=1}^{n}{\left({\hat{a}}_{ij}-a_{ij}\right)}^{2}}{\sum_{i=1}^{m}\sum_{j=1}^{n}a_{ij}{}^{2}}\right)$$

where *m* and *n* represent the number of columns and rows of the mixing matrix A, respectively, ${\hat{a}}_{ij}$ and *a_ij_* represent the elements of the mixing matrix **A** and the estimated mixing matrix $\hat{A}$ in the *i*-th row and *j*-th column, respectively. The smaller the NMSE value is, the more accurate the estimated mixing matrix is:

(24) $$ang(a,\hat{a})=\frac{180}{\pi }\cos^{-1}\left(\frac{a^{T}\hat{a}}{\left\|a\right\|\cdot \left\|\hat{a}\right\|}\right)$$

where **a** is the column vector of the original mixing matrix **A** and $\hat{a}$ is the column vector of the estimated mixing matrix $\hat{A}$. The smaller the value of *ang* is, the more accurate the estimated mixing matrix is.

The above two mixing matrix evaluation criteria are used to evaluate the estimation accuracy of the mixing matrix between the traditional K-means method and the proposed method. The results are listed as follows.

Table 2 shows that the proposed method has the smallest NMSE value and the smallest angular deviation in each column, indicating that the proposed method is superior to the traditional method in the mixing matrix estimation.

**Figure 9:**
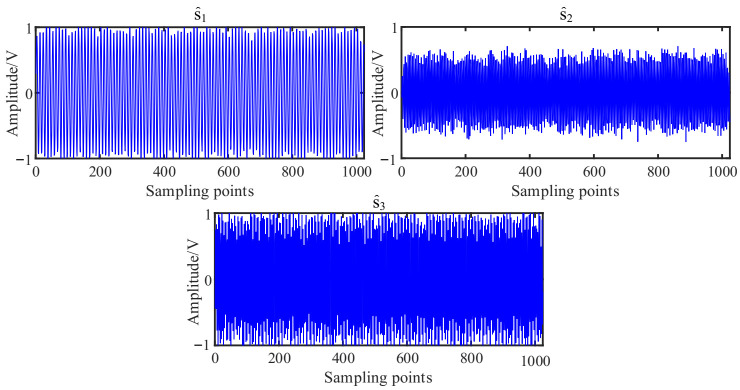
Estimated source signals by the K-means.

**Figure 10:**
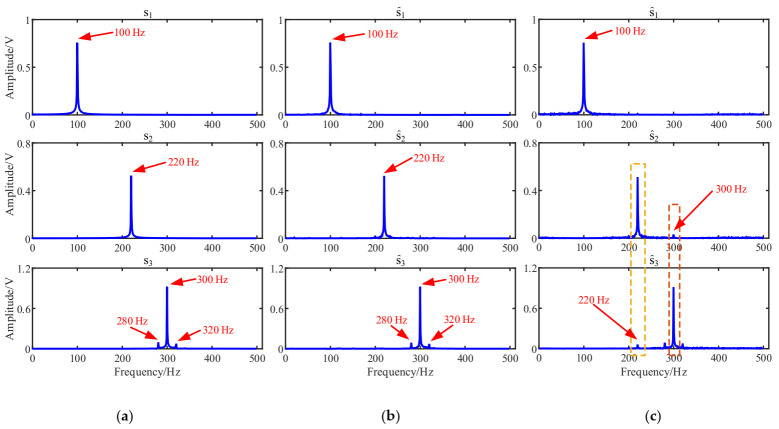
Comparison of the estimated source signals in the frequency domain: (**a**) Source signals; (**b**) The proposed method; (**c**) K-means.

The mixing matrix estimation is the most important step in UBSS, determining the performance of source recovery. After obtaining the mixing matrix, the source signals are recovered in the same way as in Section 4.1. The estimated sources are shown in Figure 9 and their Fourier spectrums are given in Figure 10c.

Comparing the estimated source signals in Figure 2a, Figure 8a and Figure 9, there is no obvious difference between these signals in terms of time domain waveforms. However, when comparing with their Fourier spectrums shown in Figure 10, we can find that the characteristic frequency of ${\hat{s}}_{2}$ and ${\hat{s}}_{3}$ in Figure 10c are disturbed by 300 Hz and 220 Hz, respectively. On the contrary, the proposed method estimated Fourier spectrums shown in Figure 10b are more similar to those of source signals in Figure 10a. Therefore, the results showed that the proposed method could outperform the traditional K-means algorithm.

To qualitatively measure the performance of the estimated source signals by the proposed method, the correlation coefficient and mean squared error (MSE) are used in this study. The correlation coefficient between the source signals and the estimated source signal is calculated by:

(25) $$R=\sum_{i=1}^{n}\left\{\frac{[s_{i}(t)-{\bar{s}}_{i}(t)]{\left[{\hat{s}}_{i}(t)-{\bar{\hat{s}}}_{i}(t)\right]}^{T}}{{\left\|s_{i}(t)-{\bar{s}}_{i}(t)\right\|}_{2}{\left\|{\hat{s}}_{i}(t)-{\bar{\hat{s}}}_{i}(t)\right\|}_{2}}\right\}$$

where ${\bar{s}}_{i}$ and ${\bar{\hat{s}}}_{i}$ denote the means of the signal *s_i_* and ${\hat{s}}_{i}$, respectively, and *n* is the number of the sources. The MSE is defined as follows:

(26) $$\mathrm{MSE}=10\log_{10}\left(\frac{1}{n}\sum_{i=1}^{n}\underset{\delta }{\min}\frac{{\left\|s_{i}-\delta {\hat{s}}_{i}\right\|}_{2}^{2}}{{\left\|s_{i}\right\|}_{2}^{2}}\right)$$

where ${\hat{s}}_{i}$ denotes the estimation of the source signal *s_i_*, and *δ* is a scalar reflecting the scalar ambiguity.

Table 3 shows the performance comparison of the source recovery with the evaluation criteria of correlation coefficients and MSE. As can be seen in Table 3, the correlation coefficient of each estimated source signal is larger than that of the traditional K-means. The MSE of the recovered sources by the K-means and the proposed method are −14.8696 dB and −22.8119 dB, which indicates that the recovered error by the proposed method is smaller. The above results validate that the improved K-means algorithm is superior to the traditional K-means. Moreover, as revealed in Table 2 and Table 3, the accuracy of the source signals recovery greatly depends on the estimation accuracy of the mixing matrix. Therefore, the mixing matrix estimation plays a more decisive role in the UBSS.

### 4.3. Experiment and Discussion

The reciprocating compressor has been widely applied in the petroleum and chemical industry, and their operating state and security are thought to be challenging research subjects. In this paper, a 2D12-70/0.1~13 double-acting reciprocating compressor was studied with the UBSS.

**Figure 11:**
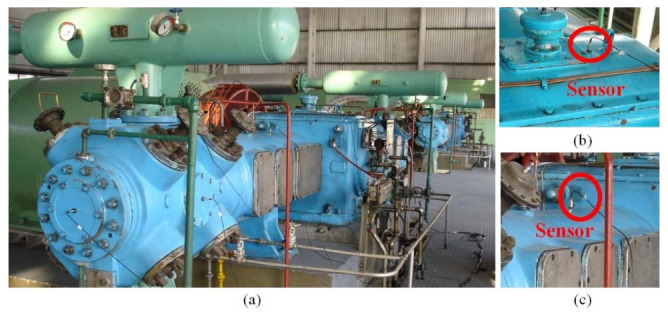
A two-stage double-acting reciprocating compressor of type 2D12: (**a**) Photos of the test bench; (**b**) Sensor on the top of the crankcase; (**c**) Sensor on the top of the crosshead guide surface.

**Figure 12:**
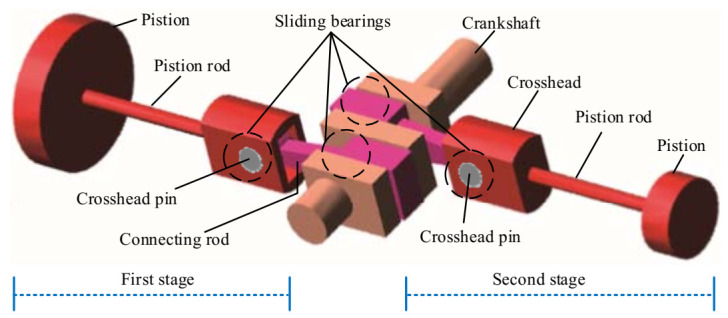
Schematic diagram of the reciprocating compressor transmission mechanism.

The shaft power of the reciprocating compressor is 500 kW, the exhaust volume is 70 m^3^/min, the exhaust pressure is 0.2746–0.2942 MPa, the piston stroke is 240 mm, the motor speed is 496 r/min, and the corresponding motor rotation frequency is 8.27 Hz. Photos of the test bench and the schematic diagram of the reciprocating compressor transmission mechanism are displayed in Figure 11 and Figure 12, respectively. As shown in Figure 12, the reciprocating compressor is driven by the machine to rotate the main shaft and transmit the power to the crankcase, which then drives the connecting rod, crosshead, and piston to make the reciprocating motion in the work engineering. However, due to manufacturing tolerances, defects, or after working for a while, the sliding bearings between the big end of the connecting rod and the crankshaft, and the small end of the connecting rod and the crosshead are susceptible to wear because of the long-term action of the reciprocating force, the bearing clearance is too large, resulting in equipment failure. Therefore, the fault diagnosis of the reciprocating compressor is carried out by combining UBSS and refined composite multiscale fuzzy entropy (RCMFE) [47].

**Figure 13:**
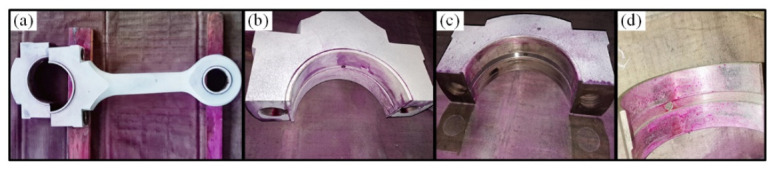
Photos of connecting rod: (**a**) Connecting rod; (**b**) Normal state; (**c**) Big end fault bearing; (**d**) Bearing bush.

**Figure 14:**
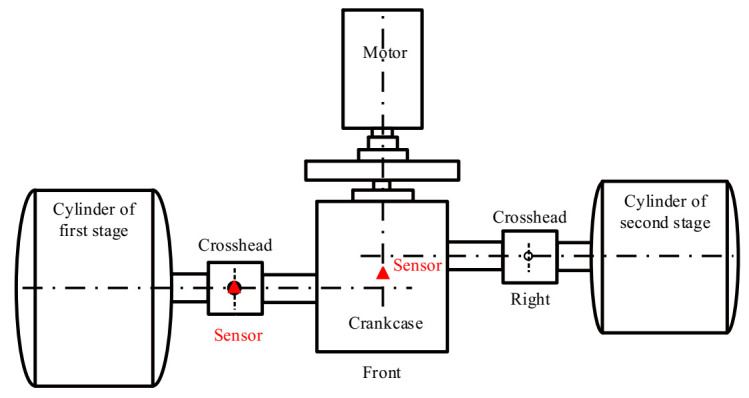
The structural draw of reciprocating compressor and measuring points.

**Figure 15:**
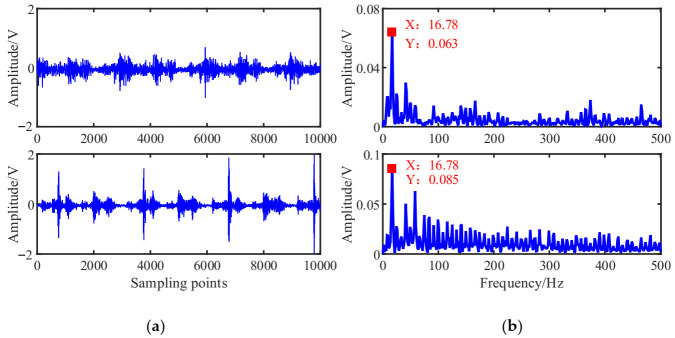
Mixed signals: (**a**) Waveforms; (**b**) Envelope spectrums.

Bearing bush wear is a long-term process, but if not detected and replaced early enough, it may lead to huge economic losses and even casualties. The early fault signal is weak and often drowned in noise, which makes it difficult to extract the fault characteristic information effectively. Therefore, this study takes the early weak fault signal of the bearing bush as the research object to verify the effectiveness of the proposed method, the connecting rod and its components are shown in Figure 13. After working for a while, the normal bearing bush in Figure 13d will wear out and then becomes the fault state in Figure 13c, resulting in a clearance between the journal and bearing bush, and the machine will produce abnormal sound and vibration. During the test, for the first-stage connecting rod big end fault state, two sensor measurement points were arranged on the compressor for data acquisition, located at the upper end of the crosshead slide and the top of the crankcase. The specific installation locations are shown in the red triangle in Figure 14. The vibration signals were collected with the Beijing Oriental Institute’s INV306U-6660 multi-channel intelligent data acquisition instrument. As shown in Figure 11b,c, the integrated circuit piezoelectric (ICP) acceleration sensors (CT1010LC) were installed in the red rectangular box with a sensitivity of 100 mv/g, a measurement range of ±50 g, and a frequency range of 0.5~5 kHz. The sampling frequency is 50 kHz, and the test lasts for 4 s. The time domain waveforms and envelope spectrums of the acquired mixed signal **x** = [*x*_1_,*x*_2_]*^T^* are shown in Figure 15.

Figure 15a shows that the mixed signal *x*_i_ waveform is more chaotic with no obvious characteristic information due to the mutual coupling of multiple excitation sources of the reciprocating compressor possibly. The waveform of mixed signal *x*_2_ shows an obvious periodic shock component caused by some fault possibly. In the envelope spectrums of Figure 15b, the double frequency of 16.78 Hz is obvious, which is determined by the mechanism of the reciprocating compressor. Because under the action of reciprocating inertial force, there will be two collisions in a cycle, and thus the double frequency of the envelope spectrum will have a peak.

**Figure 16:**
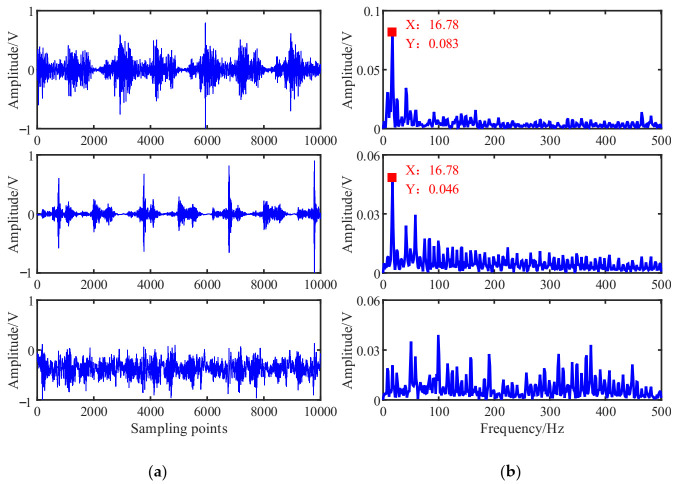
Estimated source signals: (**a**) Waveforms; (**b**) Envelope spectrums.

According to the flowchart in Figure 1, the source signals are recovered using the proposed UBSS method. The results of the estimated source signal $\hat{s}={\left[{\hat{s}}_{1},{\hat{s}}_{2},{\hat{s}}_{3}\right]}^{T}$ are shown in Figure 16. From Figure 16a, we can see that the estimated source signal ${\hat{s}}_{1}$ is a periodic excitation source, the ${\hat{s}}_{2}$ has obvious periodic impact characteristics, and the ${\hat{s}}_{3}$ is chaotic. From the envelope spectrums in Figure 16b, the first two signals have distinct double frequency components of the rotation frequency, and that characteristic frequency can not be found in ${\hat{s}}_{3}$.

**Figure 17:**
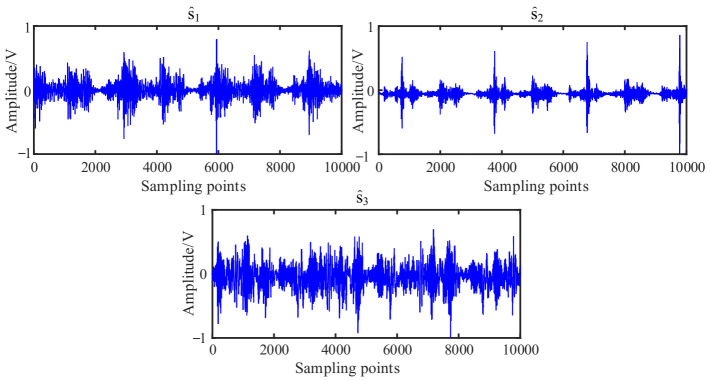
Estimated source signals by the K-means.

**Figure 18:**
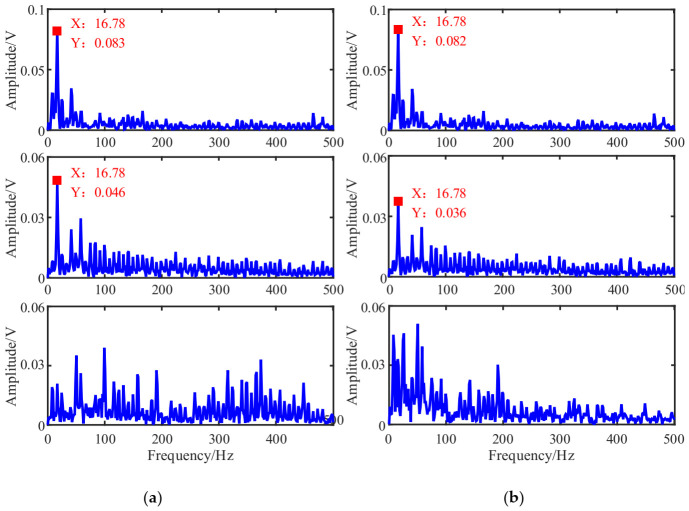
Comparison of the estimated source signals in the frequency domain: (**a**) The proposed method; (**b**) The K-means.

As a comparison, the mixing matrix was estimated by the traditional K-means algorithm, and then the sources were recovered in the same way shown. The time waveforms and their envelope spectrums were shown in Figure 17 and Figure 18b. From Figure 17, we can see that there is no obvious difference between the two methods. To facilitate comparison and analysis of the effects of the proposed method and the K-means in the frequency domain, the envelope spectrums in Figure 16b were put together with that of K-means in Figure 18.

Comparing with Figure 18a,b, the first two signals estimated by the K-means have lost some amplitude information, resulting in their characteristic frequencies being interfered with by other components, especially the signal ${\hat{s}}_{2}$ is submerged in other frequency components, and the ${\hat{s}}_{3}$ is also chaotic. Therefore, this suggests that the proposed method could outperform the contrast method in feature extraction.

Different from the fault diagnosis of the rotating machinery, the type of the estimated source signals in the reciprocating compressor can not be identified from the envelope spectrums because of the complex structure of reciprocating compressors, various forms of component movements (e.g., reciprocating and rotating movements), and the multi-component coupled signals of vibration signals. Therefore, the fault diagnosis of the reciprocating compressor is performed by comparing the collected signals with those in a standard database, which is obtained by long-term monitoring in the laboratory, serving as a standard state for fault decisions.

**Figure 19:**
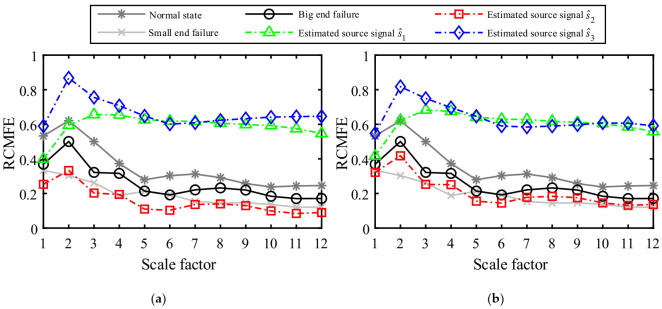
RCMFE analysis of the estimated source signals by different method. (**a**) The proposed method; (**b**) The K-means.

As a nonlinear dynamical method, information entropy can measure the degree of uncertainty of things and portray the complexity of systems, which is widely used in machinery fault diagnosis [5,48,49]. Therefore, the RCMFE-based information entropy is introduced to explore the deep fault information hidden in the vibration waveform. Firstly, the RCMFE values of the normal state signal, the first-stage connecting rod big end fault signal, and the first-stage connecting rod small end fault signal in the standard database are calculated, as shown by the solid lines in Figure 19. From Figure 19, the curves of the three states are clearly distinguished. Under normal conditions, the entropy of the collected signal is large because of the great unpredictability of the system operating state and the small self-similarity of the vibration signal. The malfunction of the equipment component generates a periodic shock, a strong self-similarity of the vibration signal, a low degree of confusion, and a small entropy value. Since the failure mechanism of the connecting rod big end and the connecting rod small end are different, their entropy values are not the same in Figure 19. The dotted lines in Figure 19a represent the RCMFE values of the estimated source signals in Figure 16a, where the blue curve has the largest entropy value, indicating the greatest degree of confusion. ${\hat{s}}_{3}$ is determined as the environmental noise with the analysis of the source signal vibration waveform estimated in Figure 16a. The green entropy curve has a low entropy value at the first two scales, while its entropy value is close to that of the noisy signal ${\hat{s}}_{3}$ at most scale factors. Further analysis of the estimated source signal combined with the Figure 16a suggests that the estimated source signal ${\hat{s}}_{1}$ is a signal coupled with multiple excitation source components. The red entropy curve under different scale factors *τ* displays the same trend as the black curve in the standard database, indicating that both curves contain the same fault characteristic information and ${\hat{s}}_{2}$ is a connecting rod big end fault. However, the red curve has a lower entropy value than the black curve because the signal contains other excitation sources such as noise due to the influence of the transmission path and other factors in the acquisition process. Besides, the UBSS can filter out some noise and excitation sources in the source signals separation process, making the estimated source signal ${\hat{s}}_{2}$ purer so that its disorder degree is lower and entropy value is smaller than the big end fault signal in the standard database, but the overall trend is still consistent with the big end fault entropy curve. As a comparison, the results by the K-means method were also calculated shown in Figure 19b. From Figure 19a,b, we can find that the results of the two methods are basically consistent. However, the entropy values of the estimated signal ${\hat{s}}_{2}$ by the K-means are larger on all scales, and its disorder degree is greater than that of ${\hat{s}}_{2}$ by the proposed method, indicating that it is disturbed by noise, which is consistent with the previous analysis results in Figure 18b.

## 5. Conclusions

This paper proposed a two-stage clustering method by combining hierarchical clustering and K-means to estimate the mixing matrix from their instantaneous mixtures in underdetermined systems. Our method can remove some outliers existing in the observed signals and then obtain the accurate initial clustering centers when compared with the K-means whose initial clustering centers are randomly generated.

The simulation analysis showed that the NMSE value of the estimated mixing matrix by the proposed method was smaller than that of the traditional K-means and the correlation coefficient of the estimated source signal was larger, indicating the proposed method could outperform the traditional K-means. The application of reciprocating compressor showed that the improved UBSS method by combining RCMFE could accurately extract fault features and identify the fault location. Although the proposed method can improve the accuracy of the mixing matrix estimation, there are still some limitations. The two-stage clustering is more time-consuming than other single clustering algorithms. And the proposed method is only employed in the underdetermined mixture system, where at least two sensors are needed. In future work, we will focus on the sparse representation of sources and applying fewer samples to cluster and reduce the computing time.

## Figures and Tables

**Table 1:** Eigenvalue of the $x_{imf}$.

**SVD**	$\lambda_{1}$	$\lambda_{2}$	$\lambda_{3}$	$\lambda_{4}$	$\lambda_{5}$	$\lambda_{6}$	$\lambda_{7}$	$\lambda_{8}$	$\lambda_{9}$
**Value**	0.4985	0.3049	0.0017	0.00086	0.00014	0.00005	0.00004	0.00004	0.00002

**Table 2:** Angular differences and NMSE of K-means and the proposed method.

Methods	Angular Difference			NMSE (dB)
	$ang(a_{1},{\hat{a}}_{1})$	$ang(a_{2},{\hat{a}}_{2})$	$ang(a_{3},{\hat{a}}_{3})$	
K-means	0.4578	0.8868	0.5895	−38.6513
Proposed method	0.3715	0.0620	0.0515	−48.3349

**Table 3:** Performance comparison of the estimated source signals with correlation coefficients *R* and MSE.

Methods	Correlation Coefficients *R*			MSE (dB)
	$<s_{1},\;{\hat{s}}_{1}>$	$<s_{2},\;{\hat{s}}_{2}>$	$<s_{3},\;{\hat{s}}_{3}>$	
K-means	0.9956	0.9871	0.9927	−17.8696
Proposed method	0.9981	0.9960	0.9981	−22.8119

## Data Availability

The datasets used or analyzed during the current study are available from the corresponding author on reasonable request.
